# Family functioning and problematic internet pornography use among adolescents: a moderated mediation model

**DOI:** 10.3389/fpubh.2023.1199835

**Published:** 2023-06-15

**Authors:** Liang Li, Xizhou Wang, Shaoyue Tang, Jianfeng Wang

**Affiliations:** School of Psychology, Chengdu Medical College, Chengdu, Sichuan, China

**Keywords:** family functioning, problematic internet pornography use, self-esteem, need to belong, adolescents

## Abstract

**Background:**

In recent years, the issue of problematic Internet pornography use (PIPU) among adolescents has become increasingly prominent, attracting widespread attention from society. Family functioning has been recognized as a protective factor for PIPU, but the underlying mediating and moderating mechanisms remain unclear. The purpose of this study is (a) to investigate the mediating role of self-esteem in the relationship between family functioning and PIPU, and (b) to examine the moderating role of the need to belong in this mediating pathway.

**Methods:**

A total of 771 high school students (*M*_age_ = 16.19, *SD* = 0.90) were surveyed using the Problematic Internet Pornography Use Scale, Family Assessment Device, Rosenberg Self-Esteem Scale, and the Need to Belong Scale.

**Results:**

Correlation analysis showed a significant negative correlation between family functioning and PIPU (*r* = −0.25, *p* < 0.001), a significant positive correlation between self-esteem and family functioning (*r* = 0.38, *p* < 0.001), a significant negative correlation between self-esteem and PIPU (*r* = −0.24, *p* < 0.001), and a significant positive correlation between need to belong and PIPU (*r* = 0.16, *p* < 0.01). Mediation analysis showed that self-esteem partially mediated the relationship between family functioning and PIPU, with a mediation effect of −0.06. Further moderated mediation analysis showed that for adolescents with higher need to belong, the mediating effect of self-esteem was stronger.

**Conclusions:**

For adolescents with high belonging needs who are at high risk for PIPU, good family functioning may have a protective effect by boosting self-esteem.

## 1. Introduction

The development of the Internet has led to an exponential increase in the number of users of online pornography. Pornography is familiar to both men and women, with research showing that watching pornography is the most addictive activity on the internet (1). The latest research estimates that the prevalence of lifetime pornography consumption is around 92–98% in men and 50–91% in women (2). Most people who watch pornography do so for entertainment purposes and do not experience serious problems. However, a small minority of pornography viewers exhibit problematic internet pornography use (PIPU), with rates of 1–7% for women and 3–10% for men (3–5). Due to the similarities between PIPU and substance addiction in terms of occurrence and development mechanisms (6, 7), PIPU is also referred to as online pornography addiction by many scholars and is typically defined as the inability to control excessive consumption of pornography despite its serious negative consequences (8). Research has found that internet pornography use is associated with many negative effects, such as physical health problems, emotional problems, and interpersonal relationship problems (9–11). Therefore, it is necessary to better understand the risk factors and related mechanisms of PIPU in adolescents in order to provide insights for prevention and intervention efforts.

**Figure 1:**
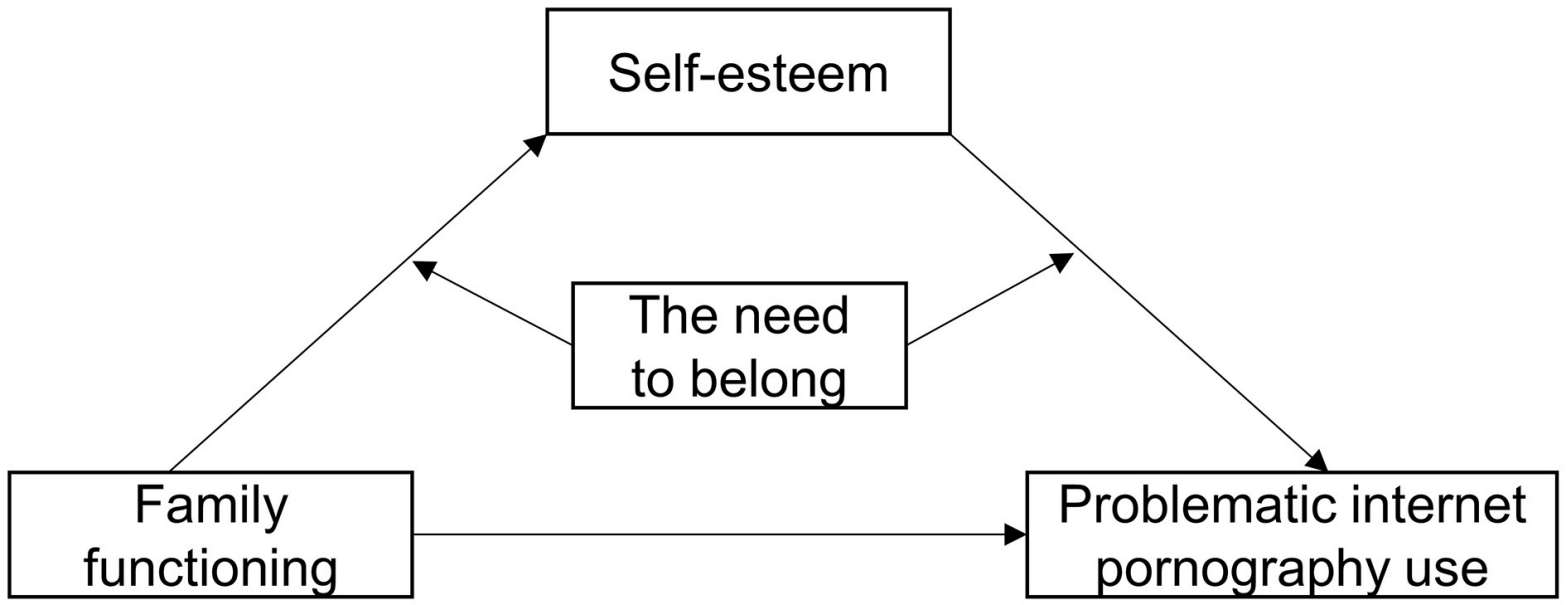
The proposed moderated mediation model.

The family, as a microsystem for personal development, is the most direct and specific microenvironment that affects psychological development and has a direct and profound impact on individual development (12, 13). A number of studies have found that family functioning has an important impact on adolescent PIPU, with good family functioning (such as parental love and care, effective communication, etc.) effectively reducing adolescent PIPU, while the breakdown of family functioning (such as lack of communication, low commitment to the family, poor parent-child relationships, etc.) significantly increases the proportion of adolescents participating in online pornography (14–16). Although previous studies have examined the relationship between family functioning and adolescent PIPU, the mediating (how family functioning is related to PIPU) and moderating (when this relationship is most closely linked) mechanisms between the two are still not well understood. Exploring the internal impact mechanism between family functioning and PIPU is of great significance for enhancing our understanding of PIPU and developing effective intervention methods. Therefore, this study constructed a conceptual model (Figure 1) with adolescents as the sample, in which self-esteem plays a mediating role between family functioning and PIPU, and the mediating path of self-esteem is moderated by the need for belonging.

As is well known, family functioning is related to self-esteem, and a large body of research shows that family functioning can predict the level of self-esteem in adolescents. For example, high levels of family intimacy and emotional expression are significantly positively correlated with high levels of self-esteem in adolescents, while denial and rejection in family relationships can lead to feelings of inferiority and helplessness, resulting in lower levels of self-esteem in individuals (17). According to Davis's cognitive-behavioral model of pathological internet use, low self-esteem is one of the risk factors for addiction (18). Some studies have reported a correlation between the use of pornography and low self-esteem, finding a positive correlation between PIPU and lower general self-esteem (19), as well as sexual self-esteem (20). Similarly, Borgogna et al. (21) found that men with low self-esteem are particularly prone to being attracted to pornography and exhibit more symptoms of PIPU. In summary, self-esteem may play a mediating role in the relationship between family functioning and PIPU, but there has been no research to date that has examined this.

Although family functioning may affect adolescent PIPU through the mediating role of self-esteem, adolescents' sensitivity to family functioning and self-esteem may differ, so it is necessary to further investigate the moderating factors of family functioning. The need to belong is a basic human motivation that has a significant impact on individuals' emotions, cognition, and behavior (22). Individuals with a high need for belonging have a higher motivation to obtain satisfactory interpersonal relationships (23). Given that an important goal of adolescent internet pornography viewing is to cope with negative emotions and alleviate feelings of loneliness (24–26), it is reasonable to hypothesize that adolescents with a high need for belonging will view internet pornography more frequently, thereby increasing the risk of PIPU. In addition, the need for belonging may moderate the indirect relationship between family functioning and adolescent PIPU. The organism-environment interaction model suggests that individuals with different personality traits react differently to similar environments, and the dynamic changes in personality traits and environment help individuals to adapt psychologically and socially (27). Family functioning, as an interpersonal or environmental factor, can only explain a small part of the differences in individual behavior. Without relevant information about individual personality traits, it is difficult to clearly explain whether family functioning will be a strong predictor of behavior. Therefore, the need for belonging can be regarded as a personality trait to better explain the relationship between family functioning and adolescent PIPU.

In summary, this study tested a moderated mediation model with adolescents as the sample. The study hypothesized that: (a) good family functioning would enhance adolescents' self-esteem, and thereby reducing PIPU. That is, self-esteem plays a mediating role in the relationship between family functioning and adolescent PIPU. (b) the need for belonging positively predicts PIPU. Meanwhile, the indirect relationship between family functioning and PIPU through self-esteem will vary depending on the level of the need for belonging. For individuals with a high need for belonging, the mediating path of self-esteem will be stronger.

## 2. Methods

### 2.1. Participants

The target schools were selected using a convenient sampling method. In two ordinary high schools located in Sichuan Province in western China, the cluster random sampling method was used to select three classes from each grade from 10th to 12th grade. Out of the 814 high school students who received the questionnaire, 800 returned the questionnaire, resulting in a response rate of 98.28%. In order to ensure data accuracy, strict screening was conducted on the collected questionnaires, excluding those with missing responses, straight-line responses (where the same answer is given for every question, such as “1, 1, 1, 1, 1…”), and pattern responses (following a certain artificial rule, such as “1, 2, 3, 4, 5, 1, 2, 3, 4, 5…”). After invalid questionnaires were removed, 771 valid questionnaires were obtained (94.72% effective response rate). There are 253 students in the 10th grade, 279 students in the 11th grade, and 239 students in the 12th grade. Of these, 405 were male (52.53%) and 366 were female (47.47%). The age range of the participants was 14–18 years old, with an average age of 16.19 ± 0.90 years old.

### 2.2. Measures

#### 2.2.1. Family functioning

The Chinese version of the Family Assessment Device (FAD) was used to assess family functioning (28). The scale contains 60 items and is divided into seven dimensions: problem-solving, communication, roles, affective responsiveness, affective involvement, behavior control, and general functioning. The scale was scored on a 4-point scale ranging from 1 (very like my family) to 4 (not at all like my family). The average of all items in each subscale was calculated as the total score for that subscale. The higher the total score, the worse the family functioning. To be consistent with positive thinking habits, the scoring direction of positive and reverse scoring questions was reversed in this study, so the higher the score, the better the family functioning. The FAD has been demonstrated to have good reliability and validity in Chinese adolescents (29, 30). The index of confirmatory factor analysis (CFA) showed an acceptable fit of the scale model: χ^2^/*df* = 4.28, RMSEA = 0.07, CFI = 0.87, TLI = 0.89, and SRMR = 0.05. The Cronbach's α coefficient of this scale in this study was 0.87.

#### 2.2.2. PIPU

The Chinese version of the Problematic Internet Pornography Use Scale (PIPUS) was used in this study (31). The scale consists of 12 questions, including four dimensions: distress and functional problems, excessive use, difficulty in self-control, and negative emotional avoidance. The scale uses a 6-point Likert scale ranging from 0 (never) to 5 (always). The PIPUS has been shown to have good internal consistency reliability and to be associated with hypothesized psychopathological variables in Chinese samples (32, 33). The index of CFA showed a good fit of the scale model: χ^2^/*df* = 1.35, RMSEA = 0.03, CFI = 0.98, TLI = 0.98, and SRMR = 0.04. The Cronbach's α coefficient of this scale in this study was 0.88.

#### 2.2.3. Self-esteem

The Rosenberg Self-Esteem Scale (RSES) was used to measure the participants' self-esteem levels (34). The scale consists of 10 items and uses a 4-point scoring system, with 1 indicating “strongly agree” and 4 indicating “strongly disagree”. The higher the score, the higher the level of self-esteem. The RSES has been well validated in Chinese adolescents (35). The index of CFA showed a good fit of the scale model: χ^2^/*df* = 3.62, RMSEA = 0.06, CFI = 0.97, TLI = 0.98, and SRMR = 0.02. The Cronbach's α coefficient of this scale in this study was 0.89.

#### 2.2.4. The need to belong

The Need to Belong Scale (NBS), developed by Leary et al. (36), measures individuals' needs for non-rejection, inclusion, and a sense of belonging. The scale consists of 10 items and uses a 5-point scoring system, with 1 indicating “strongly disagree” and 5 indicating “strongly agree”. The higher the score, the greater the individual's need for belonging. The NBS has been found to have satisfactory psychometric properties (23). The index of CFA showed an acceptable fit of the scale model: χ^2^/*df* = 4.19, RMSEA = 0.06, CFI = 0.90, TLI = 0.89, and SRMR = 0.04. The Cronbach's α coefficient of this scale in this study was 0.81.

### 2.3. Procedures and data analysis

Testing was conducted by class, with trained graduate students serving as the examiners. Before the test, the participants were given instructions for attention, and the completed questionnaires were collected on site. The data were analyzed using SPSS 22.0. Descriptive information and correlation matrices were calculated first. Then, Hayes' PROCESS macro was used for moderated mediation analysis (37). All continuous variables were standardized, and the interaction term was calculated from standardized scores. In addition, the bootstrapping method was used to test the statistical significance and obtain the standard error of parameter estimates.

### 2.4. Ethics

This study was conducted in accordance with the Declaration of Helsinki, and approved by the Ethical Committee of the School of Psychology, Chengdu Medical College (No. XL2022006). According to China's “Information Security Technology—Personal Information Security Specification” (Chinese National Standard: GB/T35273-2020), individuals between the ages of 14 and 18 can agree to and provide their own data or have their guardians provide it on their behalf, while individuals under the age of 14 must obtain their guardians' consent. In view of the sensitivity of the survey, not relying on parental consent can ensure anonymity and reduce sample bias that may distort the results. Therefore, adolescents aged 14 and above in this study signed informed consent form by themselves.

## 3. Results

### 3.1. Control and testing of common method bias

Common method bias was controlled through methods such as anonymous answering, reverse scoring, and different instructions for different questionnaires. Harman's single-factor test was used for principal component analysis, and the results showed that there were 28 factors with eigenvalues >1. The first factor accounted for 13.84% of the variance, which was < 40%, indicating that there was no serious common method bias in this study.

### 3.2. Correlation analysis

Descriptive statistics and Pearson correlation analysis were conducted, and the results are shown in Table 1. As expected, family functioning was significantly positively correlated with self-esteem and significantly negatively correlated with PIPU. Self-esteem was significantly negatively correlated with both belongingness need and PIPU. The need to belong was significantly positively correlated with PIPU.

**Table 1:** Descriptive statistics and correlations of the main variables.

**Variables**	***M***	***SD***	**Min–max**	**1**	**2**	**3**	**4**
1. Family functioning	2.73	0.22	1.85–3.48	1			
2. Self-esteem	27.52	3.44	14–36	0.38^***^	1		
3. The need to belong	33.42	5.02	14–49	−0.09	−0.14^*^	1	
4. PIPU	8.18	6.48	0–36	−0.25^***^	−0.24^***^	0.16^**^	1

N = 771.

PIPU, problematic internet pornography use; SD, standard deviation.

^*^p < 0.05.

^**^p < 0.01.

^***^p < 0.001.

### 3.3. Moderated mediation model testing

First, model 4 in Process was used to test the mediating effect of self-esteem between family functioning and PIPU. The results showed that, controlling for gender and age, family functioning significantly negatively predicted PIPU (*c* = −0.22, *SE* = 0.04, *t* = −6.66, *p* < 0.001). When family functioning and self-esteem were simultaneously entered into the equation, family functioning significantly positively predicted self-esteem (*a* = 0.36, *SE* = 0.03, *t* = 11.02, *p* < 0.001), and self-esteem significantly negatively predicted PIPU (*b* = −0.15, *SE* = 0.04, *t* = −3.97, *p* < 0.001). In addition, family functioning significantly negatively predicted PIPU (c′ = −0.16, *SE* = 0.04, *t* = −4.27, *p* < 0.001). The bias-corrected percentile Bootstrap test found that self-esteem had a significant mediating effect between family functioning and PIPU, with ab = −0.06, BootSE = 0.02, 95%CI = (−0.10, −0.03). The proportion of the total effect accounted for by the mediating effect was ab/(ab + c′) = 25.23%. Therefore, Hypothesis 1 was supported.

**Figure 2:**
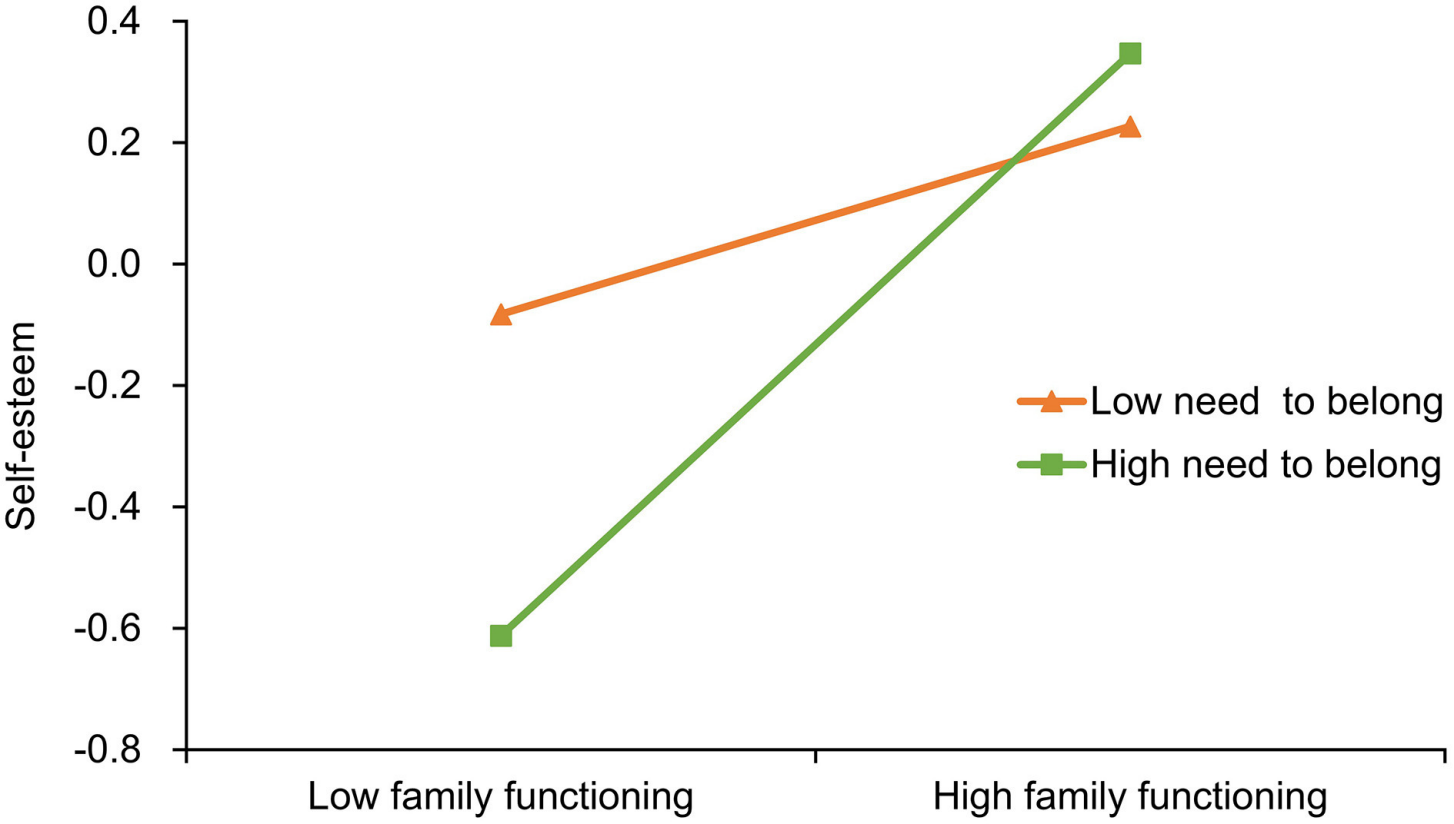
Moderating effect of the need to belong on the relationship between family functioning and self-esteem.

Furthermore, Process (model 58) was used to test the moderating effect of the need to belong on the relationship between family functioning and self-esteem (first stage) and between self-esteem and PIPU (second stage). For the first stage, the results showed that the interaction between family functioning and the need to belong significantly positively predicted self-esteem (β = 0.12, *SE* = 0.02, *t* = 5.35, *p* < 0.001), as shown in Table 2. To further explain this interaction, the need to belong was divided into high and low groups based on one standard deviation above and below the mean, and a simple slope test was conducted (38). As shown in Figure 2, for individuals with low belongingness need, family functioning significantly positively predicted self-esteem (β = 0.19, *SE* = 0.04, *t* = 2.02, *p* < 0.05); for individuals with high belongingness need, family functioning had a stronger positive effect on self-esteem (β = 0.48, *SE* = 0.04, *t* = 12.18, *p* < 0.001).

**Table 2:** Testing the moderated mediation effect of family functioning on adolescent PIPU.

**Variables**	**Model 1**				**Model 2**				**Model 3**			
	**Criterion: PIPU**				**Criterion: self-esteem**				**Criterion: PIPU**			
	**β**	***SE***	***t***	**Boot 95%CI**	**β**	***SE***	***t***	**Boot 95%CI**	**β**	***SE***	***t***	**Boot 95%CI**
Gender	−0.02	0.03	−0.39	(−0.07, 0.19)	−0.06	0.06	−0.75	(−0.15, 0.01)	0.03	0.07	0.41	(−0.11, 0.16)
Age	−0.07	0.04	−0.73	(−0.11, 0.05)	−0.01	0.04	−0.12	(−0.08, 0.07)	−0.04	0.04	−0.98	(−0.12, 0.04)
FF	−0.22^***^	0.04	−6.66	(−0.31, −0.16)	0.36^***^	0.03	11.02	(0.29, 0.42)	−0.16^***^	0.04	−4.27	(−0.23, −0.08)
NTB					−0.15^*^	0.05	−4.39	(−0.23, −0.05)	0.15^***^	0.03	4.44	(0.07, 0.24)
SE									−0.15^**^	0.04	−3.97	(−0.24, −0.06)
FF × NTB					0.12^***^	0.02	5.35	(0.07, 0.17)				
SE × NTB									−0.02	0.02	−0.69	(−0.07, 0.06)
*R^2^*	0.06				0.21				0.11			
*F*	50.51^***^				40.03^***^				15.18^***^			

N = 771.

Standardized regression coefficients are reported.

FF, family functioning; SE, self-esteem; NTB, the need to belong; PIPU, problematic internet pornography use; Bootstrap sample size = 5,000; CI, confidence interval.

^*^p < 0.05.

^**^p < 0.01.

^***^p < 0.001.

However, for the second stage, the interaction between self-esteem and the need to belong was not significant, β = −0.02, *SE* = 0.02, *t* = −0.69, *p* = 0.49. Therefore, Hypothesis 2 is partially supported.

## 4. Discussion

The impact of family functioning on adolescent PIPU has been extensively studied. However, the underlying mediating and moderating mechanisms remain unclear. This study constructed a moderated mediation model to test whether family functioning is related to adolescent PIPU through self-esteem as a mediator, and whether this indirect effect is moderated by the need for belonging. The results showed that the impact of family functioning on adolescent PIPU can be partially mediated by self-esteem, and the first half of this indirect effect is moderated by the need for belonging. In other words, for adolescents with a high need for belonging, the increase in self-esteem brought about by good family functioning is a protective factor against PIPU.

### 4.1. Mediation effect of self-esteem

The study found that good family functioning enhances adolescents' self-esteem, and self-esteem is significantly negatively correlated with adolescent PIPU. That is to say, self-esteem plays a mediating role in the relationship between family functioning and adolescent PIPU. In the first stage of the mediation model (family functioning → self-esteem), this study found that good family functioning helps to enhance an individual's self-esteem, which is consistent with self-determination theory or attachment theory (39, 40). It indicates that good family and interpersonal relationships play an important role in an individual's psychological growth (such as high self-esteem), which in turn prevents them from engaging in a series of problem behaviors such as PIPU. For the second stage of the mediation model (self-esteem → PIPU), this study showed that self-esteem negatively predicts adolescent PIPU. This finding is consistent with the cognitive-behavioral model of pathological internet use (18), which suggests that individuals with cognitive maladaptation (such as those with low self-esteem) are more likely to be addicted to the internet. In addition, this study further shows that high self-esteem is a protective factor for adolescent PIPU, which is consistent with the positive correlation between low self-esteem and PIPU found in previous studies (19–21).

### 4.2. Moderating effect of the need for belonging

This study found that the need for belonging moderates the relationship between family functioning and self-esteem, and for individuals with a high need for belonging, the predictive effect of family functioning on self-esteem is stronger. The need for belonging is a sense of social activity demand, and individuals with a strong need for belonging have a higher demand for strengthening social connections and integrating into society (41). Therefore, for these individuals, because of their higher demands for social needs and social integration, they are more sensitive to the family environment, and the impact of good family functioning on their self-esteem is greater. Previous studies have found that the need for belonging can prompt people to engage in a series of activities that may enhance social connections, and individuals with a high need for belonging may apply the internet more frequently, thereby increasing the risk of internet addiction (42, 43). This study replicated previous results and found a positive correlation between the need for belonging and PIPU. In summary, this study indicates that due to individuals with a high need for belonging have a higher susceptibility to PIPU, the protective effect of good family functioning through the increase in self-esteem is more pronounced for them. This suggests that in the prevention process of PIPU, special attention should be paid to adolescents with a high level of the need for belonging. Actively focusing on and improving their family functioning can effectively reduce the risk of PIPU.

### 4.3. Significance and limitations of the study

The results of this study are of great significance. Firstly, this study emphasizes the importance of family functioning in preventing adolescent PIPU. Negative family relationships, such as parent-child conflicts, lack of family organization, and low levels of parental support, are all related to PIPU (14–16). Especially for Chinese left-behind adolescents, neglect and lack of parental companionship may increase their risk of PIPU (44). Secondly, by establishing a mediation model, this study helps to understand how family functioning is related to adolescent PIPU and provides theoretical support for potential intervention measures, such as increasing adolescents' self-esteem level, which can effectively reduce the risk of PIPU. Finally, although belongingness is a basic need, the intensity of the need to belonging varies among individuals (23). Individuals with a high need to belong may be more susceptible to PIPU. Therefore, it is necessary to prioritize interventions for individuals with high belongingness needs in the prevention and intervention of adolescent PIPU.

There are several limitations to this study. Firstly, this study is a cross-sectional study, so causal relationships cannot be inferred. Future research should use longitudinal study designs to further validate the causal hypotheses in this study. Secondly, this study only focuses on family functioning, while other interpersonal relationships such as peer communication may also affect adolescent PIPU. Therefore, further research should consider other interpersonal relationships. Thirdly, due to the sufficient number of items already included in the questionnaire, we did not control for some background variables such as socioeconomic status (e.g., parental education level, parental occupation, and family income). Previous studies have found lower socioeconomic status to be associated with higher levels of pornography consumption (45, 46). Therefore, this potential confounds should be carefully controlled for in future research. Finally, convenience sampling was used in this study. The data was gathered from a single geographic location, which may limit the generalizability of our results to other populations. Future research should aim to improve sample representativeness so that results can be generalizable to a wider range of Chinese adolescents.

## 5. Conclusion

In conclusion, the present study demonstrates that family functioning is a protective factor for PIPU. Mediation analysis found that self-esteem may be a possible mechanism underlying this relationship. In addition, high level of need for belonging predicted PIPU in adolescents, and the mediated moderation model revealed that the protective effect of family functioning through self-esteem was stronger for adolescents with high need for belonging.

## Data availability statement

The raw data supporting the conclusions of this article will be made available by the authors, without undue reservation.

## Ethics statement

The studies involving human participants were reviewed and approved by the Chengdu Medical College Institutional Review Board. The participants provided their written informed consent to participate in this study. Written informed consent from the participants' legal guardian/next of kin was not required to participate in this study in accordance with the national legislation and the institutional requirements.

## Author contributions

LL and JW involved in study concept and design. LL, XW, and ST involved in data preparation, statistical analysis, and wrote the manuscript. JW involved in study supervision and edited the manuscript. All authors had full access to all data in the study, take responsibility for the integrity of the data, and the accuracy of the data analysis. All authors contributed to the article and approved the submitted version.
